# Peroxisome proliferator-activated receptor gamma modulation and lipogenic response in adipocytes of small-for-gestational age offspring

**DOI:** 10.1186/1743-7075-9-62

**Published:** 2012-06-22

**Authors:** Jennifer K Yee, Wai-Nang Paul Lee, Michael G Ross, Robert H Lane, Guang Han, Juan Vega, Mina Desai

**Affiliations:** 1 Department of Pediatrics, Division of Endocrinology, Los Angeles Biomedical Research Institute at Harbor-UCLA Medical Center, David Geffen School of Medicine at UCLA, 1000 West Carson Street, Harbor Box 446, Torrance, CA 90509, USA; 2 Department of Obstetrics and Gynecology, Los Angeles Biomedical Research Institute at Harbor-UCLA Medical Center, David Geffen School of Medicine at UCLA, 1000 West Carson Street, Harbor Box 446, Torrance, CA 90509, USA; 3 Division of Neonatology, University of Utah School of Medicine, Williams Building, P.O. Box 581289, Salt Lake City, UT 84158, USA

**Keywords:** Adipocyte, Small-for-gestational age, Rosiglitazone, PPARγ, Stable isotope, Fatty acid, Adipogenesis

## Abstract

**Background:**

Small-for-gestational age (SGA) at birth increases risk of development of adult obesity and insulin resistance. A model of SGA rat offspring has been shown to exhibit increased adipose tissue expression of a key adipogenic transcription factor, peroxisome proliferator-activated receptor gamma (PPARγ), and increased fatty acid de novo synthesis during the nursing period, prior to onset of obesity. PPARγ agonists have been studied for potential use in the prevention of insulin resistance. Moreover, SGA adipocytes exhibit age-dependent differences in lipogenesis as mediated by PPARγ. The effects of PPARγ modulators on lipogenic gene expression and de novo lipogenesis on the age-dependent changes in SGA adipocytes are not known. The objectives of this study were: 1) to determine the adipogenic and lipogenic potential in SGA adipocytes at postnatal day 1 (p1) and day 21 (p21), 2) to determine how the PPARγ activator- and repressor-ligands affect the lipogenic potential, and 3) to determine the fatty acid metabolic response to PPARγ activator-ligand treatment.

**Methods:**

Primary adipocyte cultures from p1 and p21 SGA and Control male offspring were established from a known maternal food-restriction model of SGA. Cell proliferation and Oil Red O (ORO) staining were quantified. Adipocytes were treated with increasing doses of rosiglitazone or bisphenol-A diglycidyl ether (BADGE). PPARγ and SREBP1 protein expression were determined. De novo lipogenesis with rosiglitazone treatment at p21 was studied using 50% U^13^C-glucose and gas chromatography/mass spectrometry.

**Results:**

At p1 and p21, SGA demonstrated increased cell proliferation and increased ORO staining. At p21, SGA demonstrated increased lipogenic gene expression and increased glucose-mediated fatty acid de novo synthesis compared with Controls. In response to rosiglitazone, SGA adipocytes further increased glucose utilization for fatty acid synthesis. SGA lipogenic gene expression demonstrated resistance to BADGE treatment.

**Conclusions:**

SGA adipocytes exhibit an enhanced adipogenic and lipogenic potential in early postnatal life. By p21, SGA demonstrated resistance to PPARγ repressor-ligand treatment, and selective response to high dose PPARγ activator-ligand treatment in adipogenic and lipogenic gene expression. p21 SGA adipocytes revealed increased fatty acid de novo synthesis through a complex relationship with glucose metabolism.

## Introduction

Human epidemiological studies have shown that small-for-gestational age (SGA) newborns demonstrate increased risk for adult obesity and metabolic syndrome [1,2]. Animal studies have effectively replicated this association [3-5] in that maternal undernutrition during rat pregnancy results in SGA. When these SGA pups are nursed normally, they exhibit hyperphagia [6], catch-up growth, and develop adult obesity and insulin resistance [4,5]. The increased adiposity has been attributed to “programmed” upregulation of the adipogenic transcription factor peroxisome proliferator-activated receptor γ (PPARγ). SGA newborns demonstrate increased PPARγ expression prior to the onset of obesity [7]. The downstream lipid target of PPARγ is a lipogenic transcription factor, SREBP1c (sterol regulatory element binding protein 1), which in turn induces expression of lipogenic enzyme fatty acid synthase (FASN) [8]. Both SREBP1c and FASN are upregulated in SGA adipose tissue at the end of the nursing period (p21) [7]. Hence, modulation of PPARγ may provide a therapeutic strategy in preventing adiposity in SGA offspring.

As an enhancer of adipocyte differentiation and lipid accumulation, PPARγ has been studied as a target for pharmacological therapy. Rosiglitazone, which is a member of the thiazolidinedione (TZD) class of drugs, is a selective ligand of PPARγ. It has the ability to activate PPARγ, and hence induce adipocyte differentiation in cell culture models and promote weight gain in rodents and humans [9,10]. Recent studies have further shown that rosiglitazone may directly induce expression of PPARγ [11,12]. Although rosiglitazone causes weight gain, long-term treatment leads to a smaller adipocyte phenotype, suggesting changes in the metabolic pathways leading to fat accumulation [13]. While studies have demonstrated that TZDs increase free fatty acid uptake in adipocytes [14,15], the effects of TZDs on endogenous fatty acid synthesis have not been well-studied. Conversely, bisphenol-A diglycidyl ether (BADGE), a selective PPARγ antagonist, has shown the ability to prevent adipocyte differentiation *in vitro*[16]. BADGE also has the ability to interfere with rosiglitazone-mediated effects on PPARγ since both PPARγ modulators (rosiglitazone and BADGE) bind at the ligand-binding domain [16,17].

Consistent with upregulated PPARγ, SREBP1c, and lipogenic target genes, our rat model of SGA offspring exhibits increased adipose tissue fatty acid de novo synthesis as early as 3 weeks of age [3]. Thus, SGA offspring demonstrate upregulation of the adipogenesis signaling cascade and enhanced adipose tissue lipogenesis prior to development of an obese phenotype. We hypothesized that the increased adipogenic potential of SGA offspring is an intrinsic cellular response, which contributes to enhanced lipogenesis. Furthermore, we hypothesized that the enhanced lipogenesis is facilitated by increased glucose-mediated fatty acid synthesis. We addressed our hypotheses by utilizing primary adipocyte cultures from 1 day old (p1) and 3 week old (p21) SGA offspring. We further examined the effect of PPARγ modulators, rosiglitazone (activator-ligand) and BADGE (repressor-ligand) on the lipogenic targets downstream of PPARγ. To delineate the specific effects of rosiglitazone treatment in the fatty acid synthesis pathways, we studied rosiglitazone-stimulated glucose utilization toward de novo fatty acid synthesis using U^13^C-glucose as a stable isotope tracer.

## Methods

### Animal studies

Studies received approval from the Animal Care Committee at the Los Angeles Biomedical Research Institute at Harbor-UCLA and were in accordance with the American Accreditation Association of Laboratory Care.

A description of the maternal food-restriction rat model has been previously published [4]. In brief, first-time pregnant Sprague–Dawley rats (Charles River Laboratories, Hollister, CA) were housed in an animal facility with controlled 12/12 hour light/dark cycles, and constant temperature and humidity conditions. On day 10 of gestation (e10), dams were either given a standard laboratory *ad libitum* chow diet (Lab Diet 5001, Brentwood, Missouri) to yield normal size pups, or were 50% food-restricted to produce SGA pups. Dams continued the assigned diets during pregnancy and lactation. After birth, on day 1 (p1), individual pups were weighed. To standardize nursing, litters were culled to 8 pups per dam (4 males and 4 females) to include the offspring of median weights. SGA offspring were cross-fostered to *ad libitum*-fed control dams. To control for cross-fostering technique, the control pups were also cross-fostered to control dams.

Male offspring (n = 6 at each age per group) were decapitated at postnatal day 1 (p1), or given an overdose of pentobarbital (200 mg/kg intraperitoneally) at day 21 (p21), then the adipose tissue was collected. Subcutaneous adipose tissue was collected at p1 due to minimal retroperitoneal adipose tissue availability at this age. Retroperitoneal adipose tissue was collected at p21 because previous data on this model already demonstrated abnormalities in this tissue [3].

### Primary adipocyte cell cultures

Pooled adipose tissue from p1 or p21 animals was minced, and digested with collagenase type II (5000U/g) in Krebs-Ringer solution for 30 min at 37 °C, filtered through 200 μm mesh nylon filter, then centrifuged at 500 x g for 5 minutes. The cells were resuspended in high glucose (450 mg/dl) Dulbecco’s modified Eagle’s medium (DMEM) (Invitrogen) with 10% fetal bovine serum (FBS) and 1% Antibiotic-Antimycotics (Invitrogen), seeded into flasks and incubated at 37 °C with 5% CO2 until confluent. For all experiments except for the cell proliferation assay, adipocyte differentiation was induced with dexamethasone (1 μM), methylisobutylxanthine (0.1 mM), and insulin (10 μg/ml) for 10 days.

### Cell proliferation assay

Preadipocytes from p1 and p21 Control and SGA male offspring were isolated as above, and seeded (1 x 10^4^ cells) in DMEM supplemented with 10% FBS and 1% Antibiotic-Antimycotics. After 24 hours, 3-(4,5-Dimethylthiazol-2-yl)-2,5-diphenyltetrazolium bromide (MTT, from Sigma) was added to each well, then cells were incubated at 37 °C. The remaining MTT was removed, the dye crystals were solubilized in isopropanol, and the absorbance was read by spectrophotometry at 570 nm wavelength (Perkin Elmer 1420 Mulitlabel counter VICTOR^3^ spectrophotometer).

### Oil red O (ORO) staining and assay

Preadipocytes were seeded (1 x 10^4^ cells) and cells were allowed to differentiate into mature adipocytes after induction as above. Cells were subsequently fixed with 4% paraformaldehyde, stained with 0.5% ORO, mounted onto slides with Vectashield mounting medium with 4',6-diamidino-2-phenylindole (DAPI; Vector) and images (40X magnification) captured (Zeiss Axioskop 40 microscope with Axiocam HRc camera).

For quantification, adipocytes stained with ORO were dried for 1 hour at 37 °C, incubated with a fixed volume of isopropanol for 20 minutes to elute the ORO, and absorbance measured at 570 nm.

### PPARγ modulation studies

After differentiation, adipocytes at p1 and p21 were incubated in high glucose DMEM with rosiglitazone (Sigma) at 0, 1 or 10 μM concentration, or with BADGE (Alexis Biochemicals) at 0, 1 or 10 μM concentration. Untreated cells received DMSO for 24 hours. Protein was extracted by sonication in RIPA buffer with phosphodiesterase inhibitor and EDTA, then the concentration was measured using the BCA Protein Assay (Pierce). 20 μg of protein was loaded on a precast BioRad BisTris gel, then transferred to nitrocellulose membranes. Non-specific binding was blocked by non-fat milk. Primary antibodies were applied overnight, membranes washed, and species-appropriate secondary antibodies applied. Autoradiography of membranes with chemiluminescence (Pierce Super Signal West Pico Chemiluminescence kit) was carried out in a darkroom. After stripping and washing of the original membrane, a primary antibody suitable for use as an internal control was applied, followed by a secondary antibody and chemiluminescence. Densitometry was performed on the protein of interest and band intensities were normalized to those of the corresponding control. The following are the antibodies used and their respective sources: PPARγ (Affinity Bio Reagents, AB Cam), SREBP1 (Santa Cruz), FASN (Santa Cruz).

### Stable isotope fatty acid metabolism studies

Primary adipocytes from p21 male RP fat were isolated and differentiated as above. For 24 hours following differentiation, Control and SGA adipocytes received DMSO or rosiglitazone 10 μM (n = 3 each group). 50% of the glucose in the high glucose DMEM was comprised of U^13^C-glucose as a tracer. After this experimental period, cells from each flask were collected as pellets for fatty acid analysis and Western blotting.

### Fatty acid extractions

Total fatty acids (from phospholipids, triglycerides, cholesteryl esters and free fatty acids) were extracted from adipocyte cell pellets, using a method by Lowenstein et al. [18]. In summary, adipocytes were saponified in 200-proof ethanol and 30% KOH (w/v) in a 1:1 volume overnight on a 70 °C heating block. Samples were acidified with HCl, fatty acids were extracted three times with petroleum ether, then air dried. Fatty acids were derivatized as methyl esters using 0.5 N methanolic HCl, dried under a nitrogen stream, and subsequently reconstituted with hexane for gas chromatography/mass spectrometry (GC/MS) analysis.

### Gas chromatography/mass spectrometry

GC/MS analysis was carried out using a Hewlett-Packard model 5973 selective mass detector connected to a model 6890 gas chromatograph. Fatty acids were analyzed as their methyl esters. Fatty acids were separated on the gas chromatograph with a Bpx70 column (30-m length, 250-μm diameter, 0.25-μm film thickness) from SGE, Inc. (Austin, TX). The GC conditions were: helium flow rate, 1 ml/min; initial oven temperature, 150 °C; temperature was programmed to increase at 3 °C/min to a final temperature of 221 °C. The expected retention times under these conditions for palmitate, palmitoleate, stearate, oleate, and vaccenate (the major fatty acids of interest in this study) were as follows: 6.5, 7.1, 9.4, 10.0, and 10.1 min, respectively. Mass spectra of fatty acids were acquired using electron impact ionization and selective ion monitoring. Ion clusters monitored for isotopomer quantitation were mass-to-charge ratio (m/z) 270 for palmitate (16:0), m/z 268 for palmitoleate (16:1), m/z 298 for stearate (18:0), m/z 264 for oleate (18:1n-9) and vaccenate (18:1n-7). Distribution of the mass isotopomers was determined from the spectral data using a method previously described by Lee et al. [19] that corrects for the contribution of derivatizing agent and ^13^ C natural abundance to the mass isotopomer distribution of the compound of interest. Each compound of interest is composed of the sum of isotopomer peaks within a cluster. The resulting mass isotopomer distribution was expressed in molar fractions (m0, m1, m2, m3, *etc.*) corresponding to the fraction of molecules that contain 0, 1, 2, 3, …^13^ C substitutions.

### Fatty acid profile

The overall fatty acid profile was determined by calculation of the area under the respective gas chromatogram peaks for each main fatty acid of interest as a percent of total fatty acids. The percent of total for each fatty acid represents the total relative abundance (labeled plus unlabeled).

### Fraction of new synthesis (FNS) over 24 hours, acetyl-CoA enrichment, and percent glucose contribution to de novo synthesis

The FNS represents the new fatty acids produced during the 24 hour experimental period, and is expressed as a percentage of total for each fatty acid. M + 2 and M + 4 isotopomers result from U^13^C-glucose incorporation into palmitate in de novo lipogenesis, thereby allowing calculation of the precursor acetyl-CoA enrichment from the consecutive mass isotopomer ratio m4/m2, and subsequent determination of the FNS. The FNS of stearate was determined from the m6/m4 ratio to minimize potential chain elongation effects on the m4/m2 ratio. Percent contribution to de novo lipogenesis (percent contribution to de novo synthesis of palmitate, the main product of de novo lipogenesis) from glucose was calculated based on the acetyl-CoA enrichment, and 50% isotope enrichment of the medium glucose [20].

### Medium glucose determination

Glucose concentration was determined on cell culture medium using the Glucose HK Assay Kit (Sigma) according the manufacturer’s instructions, using spectrophotometry. Glucose content was calculated for the volume of medium used for each culture. Glucose consumption from the medium was then determined indirectly by subtracting the glucose content of the post-treatment medium from the glucose content of the original pretreatment medium.

### Tricarboxylic acid (TCA) cycle studies

To determine the contribution of the TCA cycle to fatty acid synthesis, the relative contributions from the pyruvate carboxylase (PC) and pyruvate dehydrogenase (PDH) pathways were studied through determination of glutamate fragment enrichment [21]. Pyruvate carboxylase (PC) contributes toward production of gluconeogenic precursors through formation of oxaloacetate (OAA). PDH activity produces acetyl-CoA which can be used for fatty acid synthesis through formation of citrate. Downstream of citrate, glutamate equilibrates with α-ketoglutarate [22], and reflects different fragment enrichment patterns based on contributions from the PC and PDH pathways. Glutamate was extracted from 1 ml samples of medium by previously published methods using ion exchange columns [21,23]. Glutamate was derivatized as its n-trifluoroacetyl-n-butylester and analyzed by GC/MS using electron impact ionization. Two fragments were identified and the Σmn enrichment (fraction of ^13^ C atoms/mole) was determined for each. The fragments were analyzed for the m2 isotopomer enrichment (m2): m/z 152, representing the C2-C4 fragment (pyruvate carboxylase), and m/z 198, representing the C2-C5 fragment (pyruvate carboxylase + pyruvate dehydrogenase). The pyruvate carboxylase/pyruvate dehydrogenase (PC/PDH) ratio was determined by calculating (m2 of 152)/[(m2 of 198)-(m2 of 152)] to evaluate for differences in glucose metabolism *via* pyruvate entrance into the TCA cycle. When entry of glucose into the TCA cycle *via* PC and PDH are equal, the PC/PDH ratio is approximately 1. A decrease in the ratio suggests a relative increase in PDH activity, while an increase in the ratio suggests increased PC activity.

### Statistical analysis

Statistical analysis was performed using SigmaStat software. Comparisons were made by ANOVA 1) within each group (Control or SGA) between the basal state and the treated states with increasing dose of PPARγ modulator, and also 2) between the two groups under the same treatment conditions. Fatty acid profile and stable isotope data were analyzed among treated and untreated Control and SGA groups by two-way ANOVA, followed by pair-wise multiple comparisons using Tukey’s test. Parametric data are presented as mean ± the standard error of the mean. Data that failed normality testing was analyzed by Kruskal-Wallis ANOVA on ranks, with pair-wise comparisons using Mann–Whitney testing. The non-parametric data (individual PC and PDH data) are therefore presented as the median with the 25-75% interquartile ranges. For comparison purposes, the PC/PDH ratio is also presented as the median with the 25-75% interquartile ranges.

## Results

### Preadipocyte proliferation and adipocyte oil red O staining

**Figure 1:**
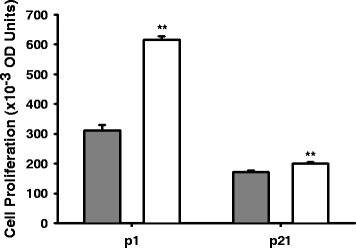
**Cell proliferation in Control and SGA offspring preadipocytes.** Gray bars represent Controls, and white bars represent SGA. Cell proliferation decreased with age in both groups. SGA demonstrated increased cell proliferation compared with Controls at both p1 and p21, **p < 0.01.

**Figure 2:**
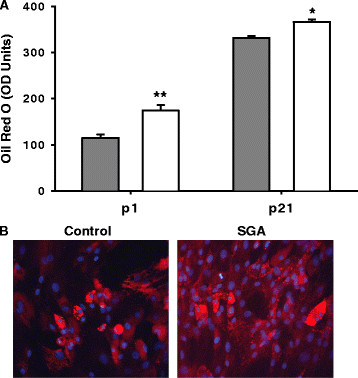
**Oil Red O (ORO) staining in Control and SGA adipocytes.** Gray bars represent Controls, and white bars represent SGA. (**A**) SGA adipocytes exhibit greater triglyceride accumulation than Controls at both p1 and p21. (**B**) SGA adipocytes at p21 demonstrate increased lipid accumulation at 40X magnification by ORO with DAPI staining. *p < 0.05, **p < 0.01.

At both p1 and p21, SGA demonstrated increased preadipocyte proliferation and increased adipocyte lipid content as compared to Controls. With age, preadipocyte proliferation decreased while adipocyte lipid ORO staining increased in both Control and SGA adipose tissue (Figures 1, 2).

### Adipogenic and lipogenic transcription factors at p1

**Figure 3:**
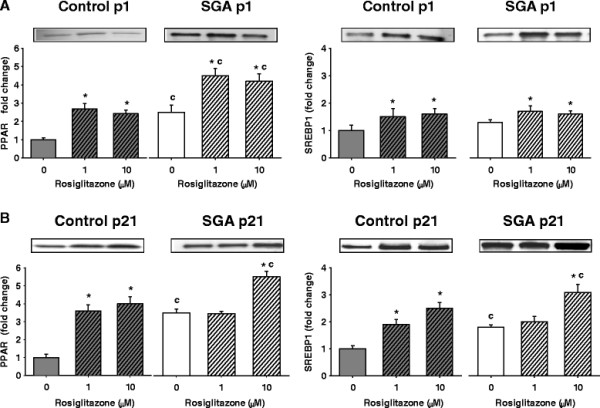
**PPARγ and SREBP1 expression by Western blotting in p1 and p21 adipocytes at baseline and with rosiglitazone treatment.** SGA maintained higher expression of PPARγ than Controls at baseline and stimulated states. (**A**) p1 Control and SGA adipocytes responded to rosiglitazone at both doses. (**B**) p21 SGA adipocytes responded only to high dose rosiglitazone. *p < 0.05 compared to untreated cells within the same group. ^c^p < 0.05 compared to Controls treated with the same dose of rosiglitazone.

At baseline, unstimulated SGA adipocytes demonstrated increased PPARγ protein expression, but comparable SREBP1 expression as compared to Controls (Figures 3A).

With rosiglitazone, Control and SGA adipocytes responded to low and high doses with marked increases in PPARγ, and moderate, though significant, increases in SREBP1 (Figure 3A). The responses to both doses were similar in each group. Furthermore, SGA adipocytes maintained higher expression of PPARγ, but similar SREBP1 expression as the Control adipocytes.

### Adipogenic and lipogenic transcription factors adipocyte phenotype at p21

At baseline, SGA adipocytes continued to show upregulated PPARγ, now accompanied by increased expression of SREBP1 (Figure 3B).

With rosiglitazone treatment, Control adipocytes responded to both (low and high) doses, demonstrating significant increases in PPARγ and SREBP1 expression (Figure 3B). In contrast, SGA adipocytes responded at only the higher dose of rosiglitazone treatment with further upregulation of PPARγ and SREBP1 expression, thereby maintaining increased expression over Controls at only this dose.

After observing that p21 SGA adipocytes were responsive to only the higher dose of rosiglitazone, stable isotope studies were undertaken during high-dose rosiglitazone treatment.

### Fatty acid metabolism and response to 10 μM rosiglitazone

#### Fatty acid profile

At baseline, SGA demonstrated a similar overall fatty acid profile as compared with Controls (Table 1). In response to rosiglitazone, Control, but not SGA adipocytes, increased the stearate percent of total, which includes preexisting and newly made stearate (Table 1).

**Table 1:** Profile of major fatty acids, at baseline and with rosiglitazone

Fatty Acids Percent of Total (%)				
	**Control**		**SGA**	
**Fatty Acid**	**Baseline**	**Rosiglitazone**	**Baseline**	**Rosiglitazone**
Palmitate 16:0	36.4 ± 1.5	37.9 ± 1.1	37.5 ± 0.7	37.7 ± 2.4
Palmitoleate 16:1n-7	3.4 ± 0.1	3.09 ± 0.04	3.4 ± 0.1	3.3 ± 0.3
Stearate 18:0	22.6 ± 0.9	29.1 ± 0.9*	20.8 ± 0.6	22.4 ± 1.4^c^
Oleate 18:1n-9	19.7 ± 0.7	17.4 ± 0.4	20.0 ± 0.3	20.1 ± 1.2
Vaccenate 18:1n-7	12.0 ± 0.5	10.4 ± 0.2	13.2 ± 0.3	12.1 ± 0.9

^C^p < 0.05 compared with Control receiving the same treatment.

*p < 0.05 compared to the untreated state within the same group.

The fatty acid percentages of total are presented as the mean ± the standard error of the mean (SEM). With rosiglitazone treatment, Controls demonstrated increased stearate abundance.

#### FNS over 24 hours

**Figure 4:**
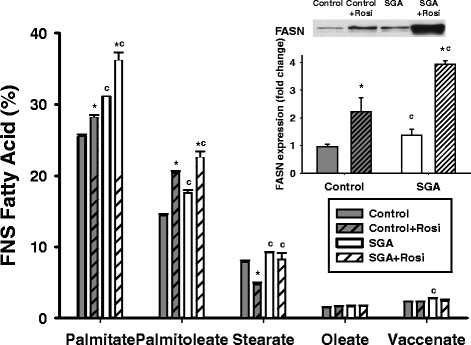
**Fraction of new synthesis (FNS) of fatty acids and FASN expression in Controls and SGA at baseline and with 10 μM rosiglitazone treatment.** The data is presented as the mean percentage or mean fold-change in expression ± the standard error of the mean. SGA demonstrated increased FNS palmitate and palmitoleate at baseline and with rosiglitazone when compared to Controls, with a similar pattern in FASN protein expression (inset). *p < 0.05 compared to untreated state within the same group, ^c^p < 0.05 compared to Controls receiving the same treatment.

At baseline, SGA had higher new synthesis (FNS) of palmitate, palmitoleate, stearate, and vaccenate over 24 hours (Figure 4).

In response to rosiglitazone (Figure 4), both Controls and SGA increased the FNS of palmitate and palmitoleate. Notably, Controls decreased the FNS stearate with rosiglitazone, while SGA did not respond. Overall, SGA maintained higher FNS rates of these three fatty acids (palmitate, palmitoleate, and stearate) than Controls. FASN expression, which was increased at baseline in SGA, also increased in response to rosiglitazone (Figure 4 inset).

#### Glucose consumption studies

At baseline, SGA adipocytes consumed more glucose over 24 hours (Table 2) than Controls. In both groups, glucose consumption was augmented by rosiglitazone treatment (Table 2), but increased more in SGA than in Controls (+interaction).

**Table 2:** Glucose consumption from cell culture medium in 24 hours at baseline or with rosiglitazone

	Glucose Consumption (mg/mL of culture medium)	
	**Control**	**SGA**
Baseline	0.99 ± 0.02	1.23 ± 0.01^c^
Rosiglitazone Treatment	1.14 ± 0.01^*^	1.45 ± 0.01*^ci^

^c^p < 0.05 compared with Control receiving the same treatment.

*p < 0.05 compared to the untreated state within the same group.

^i^ interaction with rosiglitazone.

#### Acetyl-CoA enrichment

**Figure 5:**
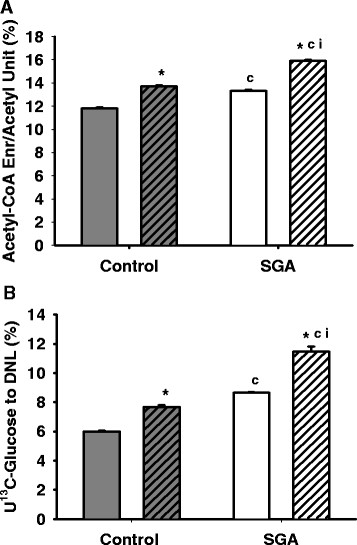
**Acetyl-CoA enrichment and U**^**13**^**C-glucose contribution to de novo lipogenesis (DNL).** Gray bars represent Controls, and white bars represent SGA. Solid bars represent adipocytes untreated at baseline, and hatched bars represent adipocytes treated with 10 μM rosiglitazone. (**A**) SGA adipocytes exhibited increased acetyl-CoA enrichment, and therefore, (**B**) increased U^13^C-glucose contribution to de novo synthesis of palmitate. There was a significant interaction between SGA and rosiglitazone for each of these measures. *p < 0.05 compared to untreated state within the same group, ^c^p < 0.05 compared to Controls receiving the same treatment, ^i^ interaction with rosiglitazone.

At baseline, SGA demonstrated increased acetyl-CoA enrichment as well as increased percentage of U^13^C-glucose contribution to palmitate de novo synthesis (Figure 5). With rosiglitazone treatment, these measures were enhanced in both groups, with SGA maintaining higher levels than Controls. Greater increases were observed in SGA (+interaction) than in Controls.

#### TCA cycle activities

To elucidate potential TCA cycle contributions to the increased acetyl-CoA enrichment, glutamate enrichment was studied to determine the relative pyruvate carboxylase and pyruvate dehydrogenase activities. SGA demonstrated increased entry of U^13^C-glucose into the TCA cycle in SGA based on the higher Σmn enrichment (fraction of ^13^ C atoms/mole) in both the glutamate C2-4 and C2-5 fragments (Table 3A). SGA demonstrated a lower PC/PDH ratio as compared with Controls (Table 3B), suggesting higher PDH activity relative to PC activity, possibly leading to increased acetyl-CoA production for lipogenesis.

**Table 3:** **Relative PC and PDH activities determined from adipocyte exposure to U**^**13**^**C-glucose**

**A.**						
	**Σmn C2-4 fragment (mean ± SEM)**			**Σmn C2-5 fragment (mean ± SEM)**		
	**Control**		**SGA**	**Control**		**SGA**
Baseline	0.056 ± 0.003		0.073 ± 0.001^C^	0.060 ± 0.003		0.084 ± 0.001^C^
Rosi Treatment	0.073 ± 0.002*		0.096 ± 0.002*^C^	0.083 ± 0.003*		0.110 ± 0.002*^C^
**B.**						
	**PC (m2 152) (median with 25-75%)**		**PDH (m2 198 – m2 152) (median with 25-75%)**		**PC/PDH (median with 25-75%)**	
**Control**	**SGA**	**Control**	**SGA**	**Control**	**SGA**	**Control**
Baseline	0.015 (0.015-0.017)	0.018 (0.017-0.018)	0.009 (0.009-0.010)	0.017 (0.016-0.018)	1.66 (1.64-1.75)	0.92 (0.90-1.04)^C^
Rosi Treatment	0.019 (0.018-0.028)	0.025 (0.025-0.026)	0.021 (0.021-0.022	0.021 (0.021-0.022)	1.10 (1.10-1.19)*	1.17 (1.14-1.18)*^i^

^C^p < 0.05 compared with Control receiving the same treatment.

*p < 0.05 compared to the untreated state within the same group.

^i^ interaction between SGA and rosiglitazone.

A) The Σmn representing the average fraction of ^13^ C atoms/molecule for the C2-4 fragment and C2-5 fragments are shown for cultures with and without rosiglitazone treatment. The data are presented as fractions of enrichment ± the SEM. Enrichment increased with rosiglitazone treatment in both Controls and SGA, while SGA maintained higher levels than Controls regardless of treatment. B) The PC and PDH pathway contributions are represented based on glutamate fragment enrichments. These data are presented as the median with the 25-75% interquartile ranges. At baseline, SGA demonstrated a lower PC/PDH ratio than Controls. With rosiglitazone, Controls decreased the PC/PDH ratio, while SGA increased it.

In response to rosiglitazone, both groups increased enrichment (Σmn) in the C2-4 and C2-5 glutamate fragments (Table 3A). Controls exhibited a decrease in the PC/PDH ratio while SGA increased it to a level similar to Controls (Table 3B), with a significant interaction between SGA and rosiglitazone. The increase of the PC/PDH ratio suggested that SGA shifted its glucose entry to be more balanced between PC and PDH, but this change could not be attributed to significant changes in either the PC or PDH enrichment beyond the changes expected based on the Σmn.

### BADGE treatment

**Figure 6:**
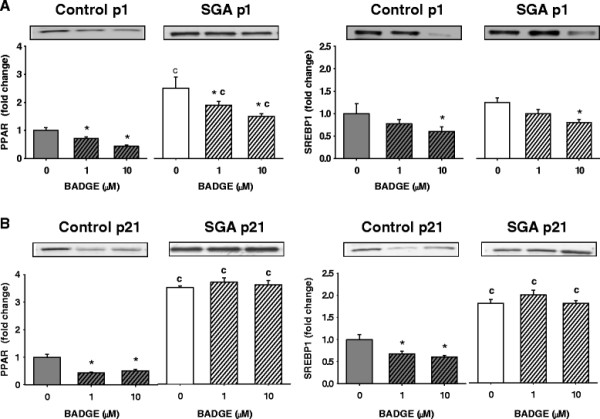
**PPARγ and SREBP1 expression by Western blotting with BADGE treatment.** (**A**) p1, (**B**) p21. At p21, SGA was resistant to both doses of BADGE. *p < 0.05 compared to untreated cells within the same group. ^c^p < 0.05 compared to Controls receiving the same dose of BADGE.

At p1, both Control and SGA adipocytes showed moderate decreases in PPARγ and SREBP1 expression at low dose BADGE, then further decreases with high dose BADGE. As before, SGA adipocytes continued to exhibit higher expression of PPARγ (Figure 6A).

At p21, Control and SGA adipocytes differed in their response to BADGE treatment. While Control adipocytes responded with downregulation of PPARγ and SREBP1, SGA adipocytes were completely unresponsive (Figure 6B). Due to the lack of response, the fatty acid metabolic studies were not repeated with BADGE.

## Discussion

This study demonstrates that SGA adipocytes are intrinsically programmed by maternal malnutrition as evident in the principal findings of: (1) enhanced adipogenesis through upregulated PPARγ expression, and (2) enhanced lipogenesis through upregulated SREBP1 and FASN expression, with increased glucose-mediated fatty acid synthesis. Importantly, SGA adipocytes are overall responsive to PPARγ activator-ligand, but are selectively responsive at p21 to the higher dose of PPARγ activator-ligand, with pathway-specific effects on fatty acid synthesis. In contrast, SGA adipocytes at p21 are unresponsive to PPARγ inhibitor-ligand. Together, these results indicate that the SGA adipocyte phenotype is programmed *in utero*, as demonstrated by enhanced preadipocyte proliferation and lipid accumulation at p1. Due to resistance to treatment at p21, therapeutic strategies to prevent the obesogenic adipocyte phenotype should be considered during the nursing period prior to onset of the resistance.

Consistent with previous studies [24,25], preadipocyte proliferation declined and adipocyte fat accumulation amplified with age in both the Control and SGA offspring. Notably, despite SGA growth-restriction at birth, the SGA adipose tissue exhibited increased preadipocyte proliferation with enhanced fat accumulation, consistent with an obese phenotype. The upregulated transcription factors (PPARγ, SREBP1) together with increased glucose-mediated fatty acid synthesis likely facilitate enhanced adipogenesis and lipogenesis in SGA offspring. Moreover, these changes are evident and persistent at postnatal age p21 in SGA offspring. Increased risk for obesity in SGA has been well-described, with SGA humans exhibiting increased visceral adiposity [26] or increased clinical features of abdominal adiposity such as waist circumference [1]. Although obesity may not manifest until adulthood, our data in nursing SGA pups demonstrating increased hyperplasia and hypertrophy along with enhanced fatty acid de novo synthesis indicates the early presence of abnormalities that may contribute toward the propensity for future fat accumulation.

The differential response to rosiglitazone revealed pathway-specific and age-specific changes in lipogenesis between Controls and SGA. Expression of adipogenic and lipogenic factors was stimulated in both groups at p1. At p21, however, Controls demonstrated increased responsiveness with dose increase, while SGA responded from its basally increased expression to only high-dose rosiglitazone. The selective response of SGA to the high-dose rosiglitazone indicates a decline in responsiveness by the end of the nursing period. Nonetheless, the decreased responsiveness of SGA at p21 indicated changes leading toward an obese phenotype as evident by the fatty acid synthesis data. Further studies are needed in offspring from p0 to p21 to determine the factors in the metabolic milieu that may ultimately lead to p21 rosiglitazone resistance.

In Controls and SGA, rosiglitazone treatment led to increased FNS of palmitate and palmitoleate, with a similar pattern of increase in FASN expression. However, the two groups demonstrated a differential response in 18-carbon stearate synthesis. The Control group increased its percent of total of stearate in response to rosiglitazone, despite decreasing the FNS stearate, suggesting decreased stearate turnover as a normal response to rosiglitazone. In contrast, SGA adipocytes did not decrease the FNS stearate in response to rosiglitazone, and maintained the new synthesis rate, suggesting abnormal resistance to modulation in the 18-carbon fatty acid synthesis pathway. The difference in FNS stearate *versus* palmitate response to rosiglitazone supports possibilities of differential regulation and/or compartmentalization of the 16-carbon *versus* 18-carbon fatty acid synthesis pathways [20]. Since the FASN expression reflected the FNS palmitate response to rosiglitazone in both groups, the stearate responses to rosiglitazone are likely mediated by a post-translational mechanism. Whether these differences in fatty acid metabolism after rosiglitazone treatment impact adipocyte function in SGA is not known from our study, but would be of interest in the setting of long-term treatment.

Rosiglitazone improves blood glucose control in humans with diabetes through increased glucose uptake by muscle and adipose tissue [27]. Therefore, we used the stable isotope U^13^C-glucose to determine the relationship between the fatty acid synthesis pathways and glucose metabolism in the adipocytes. In fact, our data demonstrate that SGA adipocytes use more glucose toward fatty acid synthesis (increased acetyl-CoA enrichment). Moreover, SGA demonstrated an augmentation of response to rosiglitazone (+interaction) possibly through a programmed increase in sensitivity to glucose abundance. Correspondingly, SGA demonstrated an increased percent contribution of U^13^C-glucose to de novo synthesis with rosiglitazone treatment. These findings led to investigations into the PC and PDH-mediated glucose entry pathways into the TCA cycle.

Analysis of the PC/PDH ratio was performed to determine how entry of glucose into the TCA cycle contributed to the increased acetyl-CoA enrichment in SGA. SGA adipocytes at baseline exhibited a lower PC/PDH ratio than Controls, suggesting that SGA adipocytes demonstrate enhanced glucose entry into the TCA cycle to produce acetyl-CoA for fatty acid synthesis *via* PDH. Rosiglitazone treatment led to a decrease in the PC/PDH ratio in Controls, supporting enhanced lipogenesis, while SGA adipocytes unexpectedly demonstrated an increased ratio. Changes in the separate PC and PDH enrichments were therefore examined relatively to changes in the Σmn. The Σmn was increased in SGA at baseline and further increased with rosiglitazone in both glutamate fragments, with corresponding trends in PC and PDH m2 enrichment. However, the SGA PC enrichment increased by 39% with rosiglitazone treatment, while the PDH enrichment increased by only 29%, resulting in a net increase in the PC/PDH ratio. An alternative explanation for the increased acetyl-CoA enrichment in our study could be increased recycling of labeled palmitate, through oxidation and reassembly of labeled acetyl units in de novo synthesis. Additional factors may also be contributing to our findings, since the turnover of glutamate is low, and the percentage of glucose contribution to palmitate comprised only up to 6-12%. The pathway of glucose utilization to produce palmitate is likely not direct [28], involving other pathways before these carbons are incorporated into fatty acids. However, this data illustrates the complex relationship that exists between fatty acid synthesis and glucose metabolism, which involve multiple metabolic pathways.

Control adipocytes responded to BADGE at both p1 and p21, with further decrease in expression of both PPARγ and SREBP1 at the high dose. In contrast, SGA adipocytes demonstrated BADGE resistance at both doses of rosiglitazone, suggesting that stimulatory factors that are present cannot be overcome by BADGE at the experimental doses. It is unknown whether even a higher dose may have been effective.

Despite the current obesity epidemic, effective strategies for the prevention or treatment of obesity and related disorders are very limited. SGA adipocytes, previously adapted to the *in utero* environment of low energy resources, suddenly experience a relative nutrient excess with normal postnatal nutrition [29]. As a result, adipocytes of SGA offspring demonstrate enhanced cell proliferation, and lipogenesis, which may allow SGA offspring to catch-up in growth. The increased adipogenesis however, leads to adult obesity and insulin resistance. TZDs are used to improve insulin sensitivity, but they have also been studied for their potential to prevent insulin resistance [30,31]. Adipose tissue is the major site of action of TZDs. Further studies are needed to define the relationship between glucose utilization for fatty acid synthesis and insulin sensitivity, in order to determine if TZDs may be efficacious in preventing insulin resistance in SGA. Studies are also needed to determine how the 16-carbon *versus* 18-carbon fatty acid synthesis pathways contribute to adipocyte phenotype in SGA, and whether differences in response of these pathways to rosiglitazone may interfere with its potential to prevent insulin resistance. If glucose-mediated lipogenesis contributes to insulin sensitivity in TZD treatment, then risks of further adipogenesis during TZD therapy must be weighed against potential benefits of glucose control.

## Conclusions

SGA adipocytes at p1 and p21 exhibit enhanced adipogenesis and lipogenesis. p1 adipocytes respond to both activator-ligand and repressor-ligand treatment. However, p21 SGA adipocytes demonstrate pathway-specific responses to PPARγ activator-ligand treatment in fatty acid synthesis. p21 SGA adipocytes are also resistant to PPARγ repressor-ligand treatment. PPARγ activator-ligand treatment enhances SGA glucose-mediated lipogenesis. The complex relationship between fatty acid synthesis and glucose metabolic pathways should be further investigated in SGA adipocytes.

## Abbreviations

SGA: Small-for-gestational age; PPARγ: Peroxisome proliferator-activated receptor gamma; BADGE: Bisphenol-A diglycidyl ether; TZD: Thiazolidinedione; ORO: Oil Red O; GC/MS: Gas chromatography/mass spectrometry; FNS: Fractional synthesis (or fraction of new synthesis); TCA: Tricarboxylic acid; PC: Pyruvate carboxylase; PDH: Pyruvate dehydrogenase; SREBP1: Sterol regulatory element binding protein 1; FASN: Fatty acid synthase; DMEM: Dulbecco’s modified Eagle’s medium; FBS: Fetal bovine serum; MTT: 3-(4,5-Dimethylthiazol-2-yl)-2,5-diphenyltetrazolium bromide; Rosi: Rosiglitazone.

## Competing interests

The authors have no competing interests to declare.

## Authors’ contributions

JKY participated in the study design, execution, analysis and interpretation of the fatty acid/stable isotope studies, and also drafted the manuscript. WPL participated in the fatty acid/stable isotope data analysis and interpretation, and contributed to manuscript preparation. MGR participated in manuscript preparation. RHL participated in manuscript preparation. GH established the adipocyte cultures, performed the MTT assay, ORO staining, and Western blotting, and contributed to manuscript preparation. JV performed Western blotting and contributed to manuscript preparation. MD participated in study design, data analysis and interpretation, and drafting of the manuscript. All authors read and approved the final manuscript.
